# Levels of Burnout and Risk Factors in Medical Area Nurses: A Meta-Analytic Study

**DOI:** 10.3390/ijerph15122800

**Published:** 2018-12-10

**Authors:** Jesús Molina-Praena, Lucia Ramirez-Baena, José L. Gómez-Urquiza, Gustavo R. Cañadas, Emilia I. De la Fuente, Guillermo A. Cañadas-De la Fuente

**Affiliations:** 1 Geriatric Center Claret, Calle Pedro Machuca N. 21b, 18011 Granada, Spain; jesusmp1995@hotmail.com; 2 Brain, Mind and Behaviour Research Center (CIMCYC), University of Granada, Campus Universitario de Cartuja S.N., 18011 Granada, Spain; edfuente@ugr.es; 3 Faculty of Health Sciences, University of Granada, Avenida de la Ilustración N. 60, 18016 Granada, Spain; jlgurquiza@ugr.es (J.L.G.-U.); gacf@ugr.es (G.A.C.-D.l.F.); 4 Faculty of Educational Sciences, University of Granada, Campus Universitario de Cartuja S.N., 18011 Granada, Spain; grcanadas@ugr.es

**Keywords:** burnout, medical area, meta-analysis, nursing, prevalence

## Abstract

Research findings concerning burnout prevalence rate among nurses from the medical area are contradictory. The aim of this study was to analyse associated factors, to determine nurse burnout levels and to meta-analyse the prevalence rate of each burnout dimension. A systematic review, with meta-analysis, was conducted in February 2018, consulting the next scientific databases: PubMed, CUIDEN, CINAHL, Scopus, LILACS, PsycINFO and ProQuest Health & Medical Complete. In total, 38 articles were extracted, using a double-blinded procedure. The studies were classified by the level of evidence and degrees of recommendation. The 63.15% (n = 24) of the studies used the MBI. High emotional exhaustion was found in the 31% of the nurses, 24% of high depersonalisation and low personal accomplishment was found in the 38%. Factors related to burnout included professional experience, psychological factors and marital status. High emotional exhaustion prevalence rates, high depersonalisation and inadequate personal accomplishment are present among medical area nurses. The risk profile could be a single nurse, with multiple employments, who suffers work overload and with relatively little experience in this field. The problem addressed in this study influence the quality of care provided, on patients’ well-being and on the occupational health of nurses.

## 1. Introduction

Stress forms part of daily life and might be considered one of the great pandemics of the 21st century [1]. In the workplace, it can affect health, personal well-being and job satisfaction, and in severe cases may provoke the appearance of burnout syndrome [2].

Burnout is composed of the following elements—emotional exhaustion (EE), depersonalisation (D) and low personal accomplishment (PA)—and appears as a result of chronic work stress [3]. The Maslach Burnout Inventory (MBI) [4] is the most commonly used questionnaire to assess the syndrome. Burnout affects workers in a growing number of professions [5] and nurses and physicians are among the most often affected [6,7]. Certain personal factors (such as gender, age, marital status, having children and personality) or external factors (such as medical records, training, work stress) may correlate with burnout development in nurses and physicians [7,8,9]. Nurses usually work in a specific medical area within a hospital, divided into units or services, according to the systems or pathologies treated. Each service has different characteristics, and these, too, can influence burnout levels [7,10,11].

The medical area (MA) incorporates the general units of a hospital complex, including services of similar characteristics and working conditions in terms of structure, organisation, work shifts, salaries, workload and type of care [12]. The only differentiating aspect within the MA would be the type of patient and the pathology treated, which determines the service providing the treatment [13].

There are conflicting research findings as to whether the appearance of burnout syndrome among MA nurses should be attributed to the type of patient [14] or to the continuous demands made on nurses by this type of hospitalisation [15], which do not usually occur in the emergency room or in primary care. The levels of burnout among MA personnel have a certain variability; although this makes the question more complex, it might be clarified by means of a meta-analysis [16,17].

Taking into account the above considerations, the present study has the following aims: to determine levels of burnout among MA nurses; to meta-analytically estimate the prevalence of EE, D and PA meta-analysis; and to determine the risk factors associated with each of these dimensions.

## 2. Materials and Methods

### 2.1. Data Sources and Inclusion Criteria

A systematic review, with meta-analysis, was carried out in February 2018, following the Preferred Reporting Items for Systematic Reviews and Meta-Analyses (PRISMA; the following are available as Supplementary Materials) [18]. The PubMed, CUIDEN, CINAHL, Scopus, LILACS, PsycINFO and ProQuest Health & Medical Complete databases were consulted.

The following inclusion criteria were applied in selecting appropriate studies for analysis: (a) there was no time restriction; (b) the studies should be written in English, Spanish or Portuguese; (c) they should be primary and quantitative; (d) they should provide data on risk factors of burnout syndrome or its prevalence; (e) they should be based on a sample of MA nurses or on a mixed sample in which the results for MA nurses are provided separately; (f) they should be conducted in the MA; (g) for the meta-analysis, they should provide independent data for prevalence for at least one of the three MBI dimensions of burnout (EE, D and PA). If the study did not use the MBI, it was included for the systematic review but not included for the meta-analysis because the domains and punctuations are not the same. No study was excluded depending on its response rate.

### 2.2. Search Strategy 

The key terms used to identify the primary studies were “burnout” combined with “nurs*” and with the type of hospital service (“internal medicine”, “cardiology”, “pneumology”, “neurology”, “nephrology”, “dialysis”, “oncology”, “haematology”, “rheumatology”, “endocrinology”). To address the entire MA, the following search formula was also used: “burnout AND nurs* AND medical wards”. The search equations were applied without any restriction, taking into account both the title and the abstract.

### 2.3. Study Selection

Independently, two members of the research team, selected the studies following the recommendations of Cooper, Hedges and Valentine [19]. For each study selected, a forward and backward search was done. In cases of disagreement between these two team members regarding the final sample of studies to be analysed, a third researcher was consulted [20]. The studies were classified by the level of evidence and degrees of recommendation from the Oxford Center for Evidence-based Medicine (OCEBM) [21].

### 2.4. Data Coding 

The data were formatted using a data coding manual, extracting the next variables: (a) authors; (b) year of publication; (c) language; (d) country where the study was done; (e) type of study; (f) sample size (nurses); (g) MA service in question (internal medicine, cardiology, pneumology, neurology, nephrology, oncology and/or haematology); (h) use of MBI (yes/no); (i) main results obtained, regarding burnout levels; (j) high presence of EE recorded; (k) high presence of D recorded; (l) low presence of PA recorded. The inter-investigator reliability of the data coding process was verified by the intra-class correlation coefficient (0.94) and Cohen’s kappa coefficient for the categorical variables (0.92).

### 2.5. Data Analysis 

The study data were analysed using the StatsDirect software (StatsDirect Ltd, Cambridge, UK). First, a sensitivity analysis was conducted. Publication bias was determined by Egger’s linear regression. The prevalence of burnout and the corresponding confidence intervals were calculated by random-effects meta-analyses. Cochran Q test and the I^2^ index were used to calculate the heterogeneity of the sample.

## 3. Results

### 3.1. Characteristics of the Study Sample

**Figure 1:**
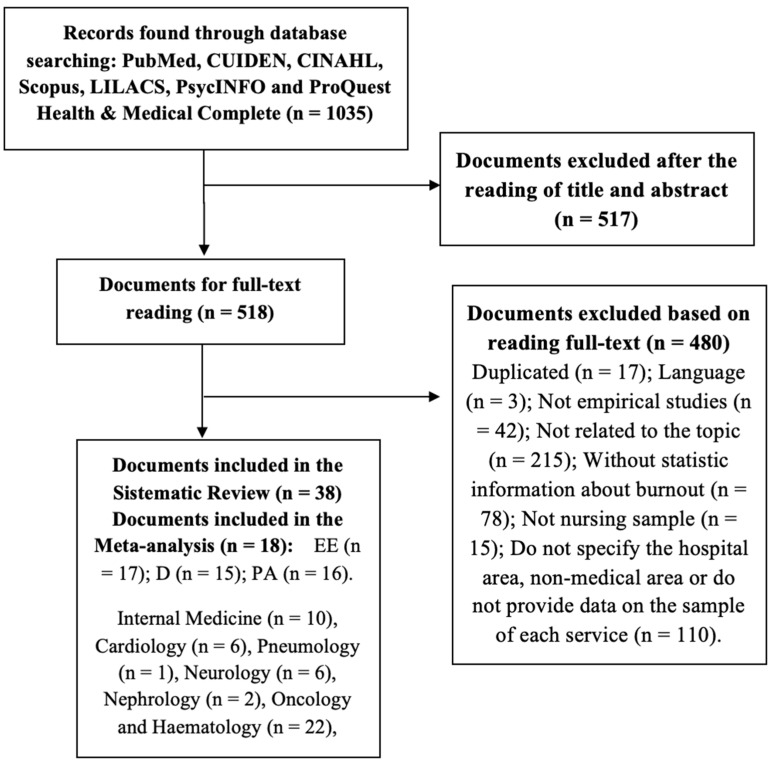
Flow diagram for the study selection process.

The search obtained n_initial_ = 1035 articles. After application of the exclusion and inclusion criteria, n = 38 remained for the systematic review and (Figure 1).

All the studies included in our analysis were cross-sectional and descriptive, with the exception of three longitudinal cohorts. The 63.16% (n = 24) of the studies used the MBI. The others studies (n = 14) were divided as follows: the Professional Quality of Life Scale (ProQOL) 7.90% (n = 3), the Spielberger State Trait Anxiety Inventory 5.26% (n = 2), the Copenhagen Burnout Inventory 2.63% (n = 1) of the studies and the rest 21.05% (n = 8) used questionnaires based on stress (Occupational Stressors Inventory, Moral Distress Scale-Revised, Nurse Stress Thermometer, etc.) and coping styles (Brief COPE, The Ways of Coping Questionnaire, Simplified Coping Style Questionnaire, etc.). Information on the level of evidence, the degree of recommendation and the main study results is shown in Table 1.

### 3.2. Main Risk Factors and Dimensions of Burnout

The majority of studies in our analysis conclude that EE is the most common dimension of burnout [22,23,26,36,39,42,44,46,48,49,50,53]. Others report a higher score for the D dimension than EE or PA among MA nurses [28,29,40,41]. Finally, a significantly greater presence of low PA has been observed in most MA services [27,34,37,38,54,57]. 

The main risk factors identified are sociodemographic. Some authors believe that younger nurses are at greater risk of burnout [26,41,43], while others hold that nurses aged over 38–40 years are more vulnerable [33,34,50]. Similarly uneven results have been reported with respect to the influence of marital status [29,34,53]. Most studies highlight the protective influence of social and family support [23,31,35,45]. The gender influence is also not clear as some studies indicate that male nurses have higher burnout levels while others say that women have higher levels or that the differences are not statistically significant [22,26,38,52].

Occupational variables associated with burnout include working night shifts [22,43,55], multiple employment [33,38], a perceived lack of work-performance recognition [25,36] and length of experience/seniority [28].

Finally, several papers observe that personality variables, together with anxiety and depression, may have a negative impact on MA services [26,28,33,36,44,46,59], although others deny that this type of variable influences the development of burnout [34] or believe its influence is slight [49].

### 3.3. Meta-Analysis of Burnout Prevalence

In total, k_final_ = 6092 nurses were included in our meta-analysis (internal medicine k = 1102, cardiology k = 244, pneumology k = 7, neurology k = 528, nephrology k = 264, oncology and/or haematology k = 3947). The meta-analysis was based on 21 samples for EE, 18 for D and 20 for low PA (see Table 1).

In our sensitivity analysis, the prevalence value obtained did not change significantly when each of the studies was eliminated from the analysis and no publication bias were detected with Egger’s test. The following values were obtained: EE = −7.13, *p* = 0.07; D = −0.69, *p* = 0.88; PA = 5.36, *p* = 0.11.

For heterogeneity, the following values were obtained by Cochran’s Q test: EE = 789.31, *p* < 0.001; D = 1162.44, *p* < 0.001; PA = 908.68, *p* < 0.001. The I_2_ index was 97.5% for EE, 98.5% for D and 97.9% for PA.

**Figure 2:**
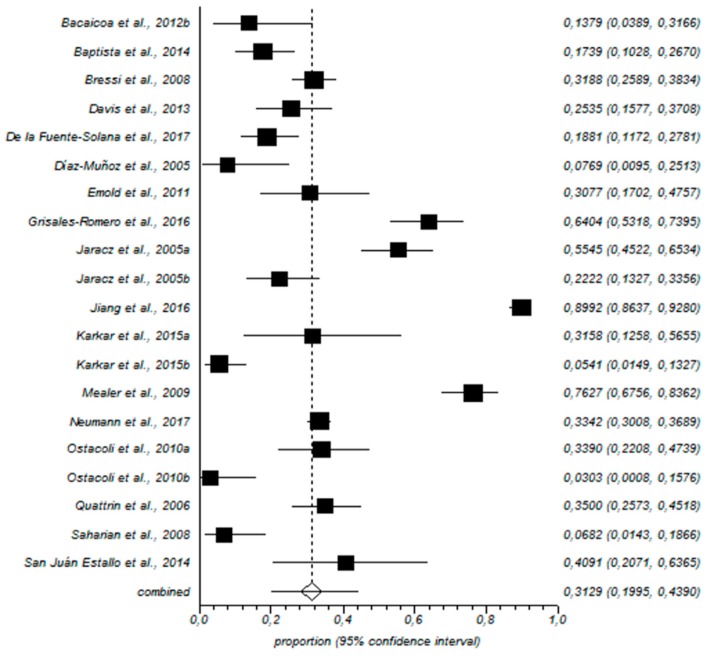
Forestplot of high EE.

**Figure 3:**
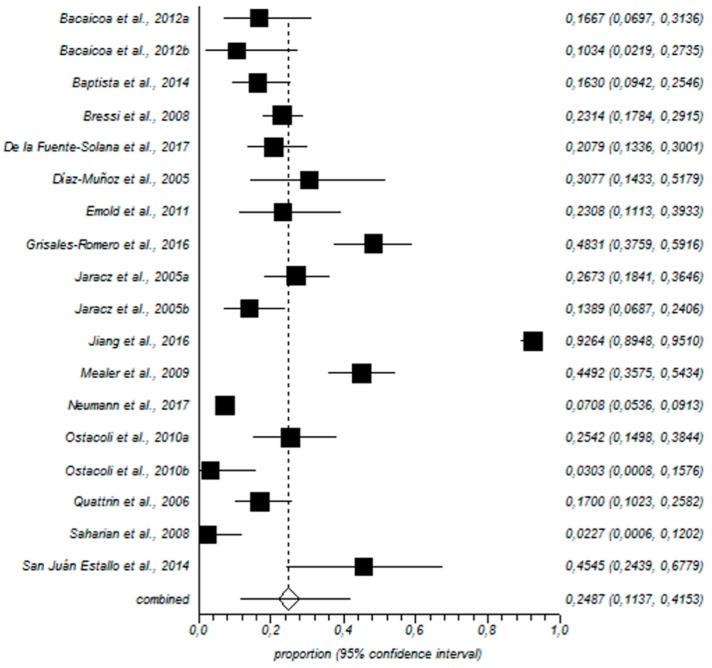
Forestplot for high D.

For prevalence, high EE was recorded among 31% of the nurses (95% CI = 19–43%), as shown in Figure 2. Figure 3 shows that high levels of D were recorded among 24% of the nurses (95% CI = 10–41%). 

**Figure 4:**
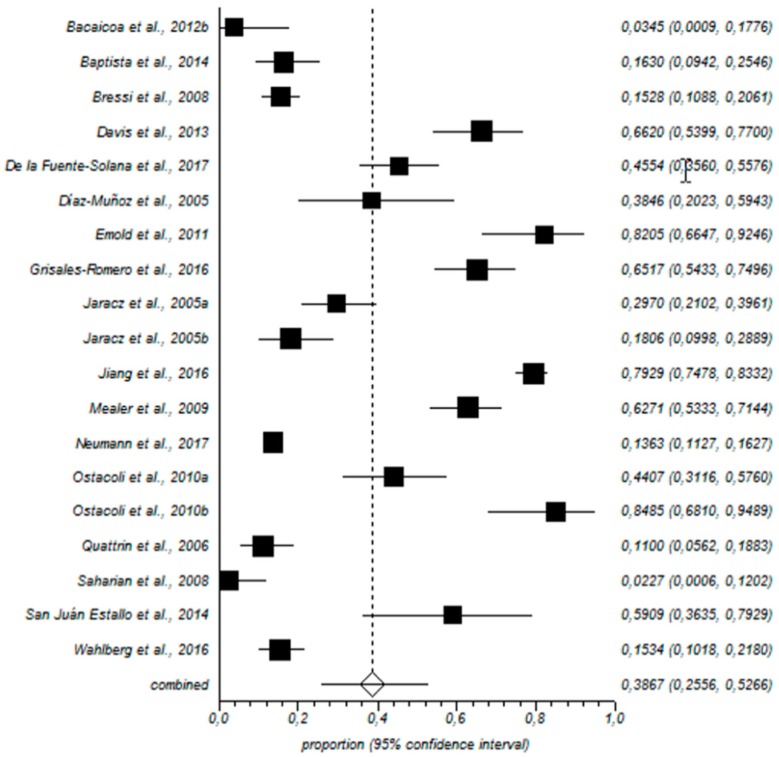
Forestplot for low PA.

Low PA prevalence rate was 38% (95% CI = 25–52%) (see Figure 4).

## 4. Discussion

To our knowledge, no previous meta-analysis studies have been done about burnout syndrome among MA nurses. We obtained a prevalence of EE of 31% among MA nurses, which is similar to other studies with emergency nurses [17] and higher to those working in primary care units [11]. Some authors have reported that EE is lower in the MA than in more specialised services [60]. However, nursing from hospital wards feel that units’ tasks (such as computers work and documentation) reduce the time that they can spend with patients what make nurses feel powerlessness and favour EE [61]. Nurses in hospital units also feel that they have too much workload, which can lead work stress, and increase EE [62].

The prevalence of D in the sample was 24%, lower than emergency nurses [17] but higher than primary care nurses [11]. In some countries, nurses from MA is responsible for a higher number of beds and patients than in other services [63], a situation that contributes to overload and consequent burnout [64]. Furthermore, visiting times for MA services are flexible, allowing the constant entry and exit of family members, which may make nurse-patient relations colder and more distant [65]. In addition, the organisational and structural distribution of the hospital service may hamper relations of trust between nurses and patients [66]. In addition, computer and documentation tasks, can make nurses feel that they cannot look after their patients [61].

The presence of low PA among MA nurses was 38%, showing that MA nurses are less accomplished that emergency or primary care nurses [11,17] and being the most affected burnout dimension. Previous studies have highlighted feelings of dissatisfaction and abandonment among MA nurses when their work is distributed impersonally, by tasks [67]. Job satisfaction is much greater when nurses feel they are providing personalised care [67]. Indeed, research has shown that establishing ties with patients and spending more time with them enhances nurses’ PA [61].

Among the occupational variables relevant to burnout among MA nurses, one that is prominent but has so far received very little research attention is that of multiple employment. Due to a lack of job security, reduced working hours and limited work availability in the public sector [68], many young MA nurses are forced to find work in both private and public institutions, a situation that is prejudicial to their health status [69] and contributes to the emergence of burnout [70].

Regarding the relation between personality variables and the different dimensions of burnout, our study obtained results comparable with those published previously [71], although the impact of responsibility can be a problem in these medical services, due to the work overload often presented, which can generate a high level of stress and hence burnout [72].

In relation to results’ applicability, nurse managers should consider that MA units where nurses have a high workload, mainly the documentary and computer work with low nurse-patient contact, tends to favor burnout [61]. Consequently, they should take measures that favor a better work environment for nurses, with fewer documentary tasks, which will allow nurses to spend more time taking care of their patients. This may increase their personal fulfillment. Also, regarding the levels of burnout, nurse manager should promote and implement different interventions to reduce burnout like orientation programs or professionals support groups [16]. Reducing and preventing burnout its negative effects on staff and patient health will be avoided [3], improving health quality and nursing care results. 

Nursing professionals should also be aware that daily tasks in a medical unit do not only include patient care [12,13]. Nurses say that they learned how to take care of patients and that documentation and computer tasks subtract their time for patients, favoring low personal accomplishment [61]. To create more realistic expectations about nursing daily tasks, the content of the nursing degree should also include more information and education about documentation and computer tasks. Another task related to nursing care is the prescription of medicines, a new competence for nursing in Europe. Medicines prescription has been already identified as a stress source in doctors due to possible errors, and it may happen the same in nurses [73]. However, it can also be a motivation source for nurses because it is a way of professional development. 

Future research should pay attention to interventions that can prevent burnout development in MA nurses and interventions that can reduce burnout suffering. For example, some interventions (such as mindfulness, meditation, resilience and coping programs) that have demonstrate to be effective for compassion fatigue and burnout among healthcare, emergency and community service workers should be taking into account for medical area nurses [74]. It would be also of great interest to analyze which personality factors are more suitable for working in MA units without developing burnout. Finally, another important thing about meta-analytic future researches is the importance of guarantee their replicability, which will be possible by including detailed information in primary research papers [75]. 

## 5. Conclusions 

MA nurses are mostly affected by low levels of PA, followed by high EE and high D. There is a greater prevalence of burnout among single persons, those in multiple employment, those who suffer work overload and those who have relatively little experience in this field.

The problem addressed in this study has impact on the quality of care provided, on patients’ well-being and on nurses occupational health. Since the MA contains most of the hospital’s long-term services, more preventive measures are needed in this area. To achieve these goals, there must be organisational, healthcare and occupational changes, based on current scientific evidence.

## Figures and Tables

**Table 1:** Characteristics of the studies included.

Authors, Country, Year	Study design	Total sample and MA	Burnout Questionnaire	EE (k)	D (k)	PA (k)	Main results	LE	DR
Álvarez-Verdugo et al., Colombia, 2013 [22]	Cross-sectional, descriptive	k = 22 GYNE k = 2 PAED k = 3 ORTHO k = 4 NEURO k = 5 IM k = 5 SRGY k = 3	MBI	-	-	-	Higher EE in the first 10 years of work and lower EE subsequently. Night work is a risk factor for burnout. Job insecurity is a risk factor for EE. Gender, age, marital status and education do not influence the appearance of burnout.	2c	B
Bacaicoa et al., Spain, 2012 [23]	Cross-sectional, descriptive	k = 71 CARDIO	MBI	13	7	25	Cardiology nurses suffer high EE and low PA. The most influential factors for burnout are the family, changes of medical service, the pressure of hospital admissions, training outside working hours, the need to work weekends and poor adherence to the interdisciplinary team.	2c	B
				4	3	1			
Baptista et al., Brazil, 2014 [24]	Cross-sectional, descriptive	k = 92 CARDIO	MBI	16	15	15	Shift work affects nurses’ quality of life and the health care provided. All three dimensions of burnout presented similar levels of prevalence.	2c	B
Basu et al., UK, 2016 [25]	Cross-sectional, descriptive	k=174 A&E k = 61 NEURO k = 45	Health and Safety Executive Stress Indicator Tool	-	-	-	Management of and participation in organisational change can reduce work stress. Lack of job recognition, together with organisational injustice, can provoke burnout in neurology nurses.	2c	B
Bressi et al., Italy, 2008 [26]	Cross-sectional, descriptive	k = 350 ONC-HAEM	MBI	73	53	35	There is a high prevalence of burnout in oncological nurses, associated with increased depression and anxiety. High levels of EE were recorded, well above those of D and inadequate PA. Age, sex, personal dissatisfaction, physical fatigue and working with demanding patients all predispose to burnout.	2c	B
Davis et al., USA, 2013 [27]	Cross-sectional, descriptive	k = 71 ONC-HAEM	MBI	18	-	47	The work environment can provoke EE and D, although low PA is the major dimension of burnout. EE is lower in younger and busier nurses. Job satisfaction is inversely associated with EE in the oncology services.	2c	B
De la Fuente-Solana et al., Spain, 2017 [28]	Cross-sectional, descriptive	k = 101 ONC-HAEM	MBI	19	21	46	D and EE are positively correlated with neuroticism, anxiety and depression and inversely with friendliness, responsibility, extraversion and openness. PA is inversely correlated with all the personality variables considered and with anxiety and depression.	2c	B
Díaz-Muñoz, Spain, 2005 [29]	Cross-sectional, descriptive	k = 26 CARDIO k = 11 ICU k = 15	MBI	2	8	10	EE was observed more frequently in female nurses, those who are single and those without children. There was greater D among nurses with less training and those without children. PA was low among single nurses and ward staff, and was the dimension most strongly affected among all respondents. The degree of burnout observed was relatively low, although only 8% of nurses reported normal scores for the three subscales.	2c	B
Duarte et al., Portugal, 2017 [30]	Cross-sectional, descriptive	k = 221 ONC-HAEM	Professional Quality of Life Scale and Interpersonal Reactivity Index	-	-	-	Nurses who are more likely to experience the negative consequences associated with providing health care (burnout and fatigue compassion) are more self-critical and have greater psychological rigidity. Moreover, they experience more personal feelings of anguish when they see others suffering and less feelings of empathy and sensitivity.	2c	B
Emery, USA, 1993 [31]	Cross-sectional, descriptive	k = 155 ONC-HAEM	Spielberger State Trait Anxiety Inventory and Pediatric Oncology Nurse Stressor Questionnaire	-	-	-	Nurses who only work in paediatric oncology for extended periods present higher levels of burnout than those who work in different areas. Specialisation and studies to obtain a Master’s degree protect against burnout. Coping styles, positive reinforcement and social support are necessary to deal with the syndrome.	2c	B
Emold et al., Israel, 2011 [32]	Cross-sectional, descriptive	k = 39 ONC-HAEM	MBI	12	9	32	EE and low PA are different experiences that can occur simultaneously. High scores were recorded for lack of PA. Communication skills, self-efficacy and cynicism are all related to the occurrence of burnout.	2c	B
Faria et al., Brazil, 2007 [33]	Cross-sectional, descriptive	k = 43 ONC-HAEM	Spielberger State Trait Anxiety Inventory	-	-	-	Oncology nurses experience high levels of anxiety and stress. The number of patients treated, the hours worked, multiple employment, age and work experience all influence the development of burnout.	2c	B
Fawzy et al., USA, 1983 [34]	Longitudinal, cohorts	k = 57 ONC-HAEM k = 12 GYNE k = 11 IM k = 11 CARDIO k = 11 SRGY k = 12	Minnesota MultiPhasic Inventory, Locus of Control Test and Work Environment Scale	-	-	-	IM nurses have less job satisfaction than oncology nurses, as well as lower PA. No statistically significant association was found between personality variables and burnout. Older, married and more experienced nurses suffer greater burnout. Social support is the main protector against the syndrome.	2b	B
Gama et al., Portugal, 2014 [35]	Cross-sectional, descriptive	k = 360 IM k = 184 ONC-HAEM k = 176 (ONC k = 48 HAEM k = 69 PALLIAT k = 59)	MBI	-	-	-	By burnout scores, there were no significant differences between hospital departments, except in palliative care, which presented lower EE and D and greater satisfaction and PA. Attitudes towards death and life, social support and length of professional experience are all protective factors against burnout.	2c	B
Gomes et al., Portugal, 2013 [36]	Cross-sectional, descriptive	k = 96 ONC-HAEM	Occupational Stressors Inventory and Brief COPE	-	-	-	Oncology nurses present high levels of EE and burnout, due to a lack of work recognition, the special characteristics of the patient and attitudes towards death. Depression and anxiety are common in oncology nurses. Active coping mechanisms should be encouraged.	2c	B
Gómez-Sánchez et al., Spain, 2009 [37]	Longitudinal, cohorts	k = 132 IM	MBI	-	-	-	Between 1998 and 2005, levels of EE fell, D remained constant and low PA worsened among IM nurses. Reducing work overload and enhancing safety would protect against burnout.	2b	B
Grisales-Romero et al., Colombia, 2014 [38]	Cross-sectional, descriptive	k = 174 IM k = 89 SRGY k = 9 ICU k = 36 CB k = 29 OTHER k = 11	MBI	57	43	58	The prevalence of burnout is higher in the study hospital than elsewhere. Male gender, multiple employment and less education are all positively associated with burnout. In IM, low PA among nurses is particularly apparent.	2c	B
Jaracz et al., Poland, 2005 [39]	Cross-sectional, descriptive	k = 173 IM k = 101	MBI	56	27	30	The level of stress influences burnout in nurses. The correlation between burnout and coping style is weak but statistically significant. EE is the burnout dimension that is most strongly affected in IM and neurology nurses.	2c	B
		NEURO k = 72		16	10	13			
Jiang et al., China, 2014 [40]	Cross-sectional, descriptive	k = 367 NEURO	MBI	330	340	291	Neurology nurses present high levels of burnout; D is the dimension that is most affected. Length of experience and holding a senior position both predispose nurses to abandon the profession and/or to suffer burnout.	2c	B
Karakoc et al., Turkey, 2017 [41]	Cross-sectional, descriptive	k = 171 NEPHRO	MBI	-	-	-	No differences were observed between the prevalence of burnout among nephrological nurses and in other hospital services. EE and D are higher in those who wish to leave the service, those lacking training and those who have difficulty in working as a team. Male gender, youth, a lack of experience and working more than 50 h a week all predispose to D. Low PA is more evident among younger nurses.	2c	B
Karkar et al., Saudi Arabia, 2012 [42]	Cross-sectional, descriptive	k = 93 NEPHRO	Modified stress and burnout questionnaires	6	-	-	Dialysis nurses present higher levels of burnout than other workers, due to the type of patients treated, the equipment employed and working in shifts. Verbal aggression by supervisors and/or patients contributes to a high prevalence of EE. Coping strategies should be developed.	2c	B
				4	-	-			
Kousloglou et al., Greece, 2014 [43]	Cross-sectional, descriptive	k = 174 PSYCH k = 25 PAED k = 48 IM k = 47 SRGY k = 54	MBI	-	-	-	An association was found between insomnia and burnout. The correlation was positive with EE and D, and negative with PA. Younger nurses, those who work more than four night shifts per month and those who work in the surgical area all experience higher levels of insomnia.	2c	B
Ksiazek et al., Poland, 2011 [44]	Cross-sectional, descriptive	k = 60 ONC-HAEM	MBI	-	-	-	Levels of EE and burnout are higher in oncology nurses than in other hospital services. Psychological variables, depression and anxiety all influence the development of burnout. Decision making and greater involvement are relevant occupational factors.	2c	B
Kutluturkan et al., Turkey, 2016 [45]	Cross-sectional, descriptive	k = 140 ONC-HEM	MBI	-	-	-	Greater resilience is associated with lower levels of burnout among oncology nurses. Coping styles, communication skills and social support all influence resilience and burnout.	2c	B
Mealer et al., USA, 2010 [46]	Cross-sectional, descriptive	k = 332 ICU k = 98 OTHER = 74 IM k = 118 PHC k = 42	MBI	90	53	74	Burnout has a dramatic effect on work-related and non-work-related perceptions. Anxiety and depression among nurses are common. MA nurses are more prone to burnout than those in other services. Of the three dimensions of burnout, EE presents the highest prevalence, followed by low PA and D.	2c	B
Nowakowska et al., Poland, 2016 [47]	Cross-sectional, descriptive	k = 405 CARDIO k = 36 NEURO k = 32 ONC-HAEM k = 24	Copenhagen Burnout Inventory	-	-	-	Organisational factors can promote professional effectiveness and efficiency, and the quality of health care provided by nurses. Those with low self-efficacy are more likely to suffer from burnout.	2c	B
Neumann et al., USA, 2017 [48]	Cross-sectional, descriptive	k = 763 ONC-HAEM	MBI	255	54	104	Burnout is moderated by variables such as discipline and stamina. Nurses presenting burnout have a greater imbalance in their work and obtain less job satisfaction. Among the dimensions of burnout, EE is the most prominent.	2c	B
Ostacoli et al., Italy, 2010 [49]	Cross-sectional, descriptive	k = 92 ONC-HAEM	MBI	20	15	26	There is a high prevalence of anxiety and depression among oncology nurses. EE is higher among hospital workers than in other institutions, while low PA is prevalent in both cases. Institutional factors are the main drivers of burnout, while personality variables have least impact.	2c	B
				1	1	28			
Quattrin et al., Italy, 2006 [50]	Cross-sectional, descriptive	k = 100 ONC-HAEM	MBI	35	17	11	Most oncology nurses present high levels of stress and burnout, due to poor organisation of the health care institution. EE is the major burnout dimension, followed by D and low PA. Women aged over 40 years, those with more work experience and those working in the oncology service are all at higher risk of burnout.	2c	B
Rodrigues et al., Brazil, 2008 [51]	Cross-sectional, descriptive	k = 77 ONC-HAEM	The Ways of Coping Questionnaire	-	-	-	The situations that predispose to burnout in oncological nurses are the deaths of patients, emergency situations and relationship problems with the nursing team. Positive coping styles are a protective factor against burnout.	2c	B
Sadati et al., Iran, 2016 [52]	Cross-sectional, descriptive	k = 371 IM	MBI	-	-	-	Burnout is associated with sociodemographic and occupational risk factors. Personal reinforcement, nursing experience and rotating shift patterns are all elements that reduce burnout.	2c	B
Sahraian et al., Iran, 2008 [53]	Cross-sectional, descriptive	k = 180 IM k = 44 SRGY k = 46 PSYCH k = 45 BURNS k = 45	MBI	3	1	1	IM nurses experience less burnout than nurses in other services. EE is the major burnout dimension. Single status is a risk factor for burnout. Different work environments have varying degrees of impact on the development of burnout.	2c	B
Sanjuán-Estallo et al., Spain, 2014 [54]	Cross-sectional, descriptive	k = 22 CARDIO k = 8 NEUMO k = 7 NEURO k = 7	MBI	9	10	13	No significant differences in burnout were observed by age or among different hospital services. Neurology nurses present the same levels of burnout as other nurses in the MA. A notable prevalence of low PA was recorded in all hospital services.	2c	B
Sehlen et al., Germany, 2009 [55]	Longitudinal, cohorts	k = 406 ONC-HAEM	Questionnaire for Ascertaining Stress on Doctors and Nurses and Global Job Satisfaction Questionnaire ad hoc	-	-	-	Nursing is the occupational area presenting highest levels of work-related stress and burnout. The work environment has a negative impact on the development of the syndrome. Low salaries, working night shifts and the nurse’s age all have a significant influence on burnout, as does long-term patient treatment.	2b	B
Sirilla, USA, 2014 [56]	Cross-sectional, descriptive	k = 73 ONC-HAEM	Moral Distress Scale-Revised	-	-	-	High levels of burnout were recorded in oncology nurses, regardless of their experience or hospital service. The higher the level of education, the lower the degree of burnout experienced.	2c	B
Wahlberg et al., USA, 2017 [57]	Cross-sectional, descriptive	k = 163 ONC-HAEM	Nurse Distress Thermometer and Occupational Coping Self-Efficacy Questionnaire for Nurses	-	-	25	Nurses who have active coping mechanisms are less subject to burnout. There is an inverse relationship between institutional support and burnout in oncology nurses. Low PA is the most significant dimension of burnout.	2c	B
Wu et al., USA, 2017 [58]	Cross-sectional, descriptive	k = 549 ONC-HAEM	Professional Quality of Life Scale	-	-	-	A healthy working environment and institutional support are both essential to nurses’ health. Improvements in the workplace can help prevent burnout and improve health outcomes for patients.	2c	B
Yu et al., China, 2016 [59]	Cross-sectional, descriptive	k = 650 ONC-HAEM	Chinese version of the Professional Quality of Life Scale for Nurses and Simplified Coping Style Questionnaire	-	-	-	Higher levels of burnout in oncology nurses were found in nurses with greater experience, those working in secondary hospitals and those with passive coping styles. The personality traits of openness and responsibility are protective against the syndrome, while neuroticism is a risk factor.	2c	B

Note: A&E: Accident and emergency; CARDIO: cardiology; CB: Childbirth; DR: Degree of recommendation, according to OCEBM; GYNE: gynaecology and obstetrics; ICU: Intensive care unit; IM: Internal medicine; k: Number of nurses sampled; LE: Level of evidence, according to OCEBM; MA: Medical area; MBI: Maslach Burnout Inventory; NEPHRO: Nephrology; NEUMO: Neumology; NEURO: Neurology; ONC-HAEM: Oncology-haematology; ORTHO: Orthopaedics; PAED: Paediatrics; PALLIAT: Palliative care; PHC: Primary health care; PSYCH: Psychiatry; SRGY: Surgery.

## Supplementary Materials

The following are available online at http://www.mdpi.com/1660-4601/15/12/2800/s1, Table S1. PRISMA 2009 Checklist.
